# Aggressive Cutaneous Squamous Cell Carcinomas Following Treatment for Graft-versus-Host Disease: A Case Report and Review of Risk Factors

**DOI:** 10.3390/dermatopathology9020015

**Published:** 2022-03-31

**Authors:** Gehan A. Pendlebury, Michelle A. Bongiorno, Jeffrey N. Lackey

**Affiliations:** 1 College of Osteopathic Medicine, Nova Southeastern University, Fort Lauderdale, FL 33314, USA; 2 Department of Dermatology, Walter Reed National Military Medical Center, Bethesda, MD 20814, USA; michelle.a.bongiorno.mil@mail.mil; 3 Department of Dermatology, Tulane University School of Medicine, New Orleans, LA 70112, USA; jlackey@tulane.edu

**Keywords:** squamous cell carcinoma, Graft-Versus-Host Disease, voriconazole, hematopoietic stem cell transplant, immunosuppressive therapy, morbilliform drug eruption

## Abstract

A 19-year-old female with a history of pre-B cell acute lymphocytic leukemia (ALL) presented with two aggressive cutaneous squamous cell carcinomas (C-SCC) in the right hand. The patient was diagnosed with pre-B cell ALL at four years of age. She underwent chemotherapy with initial remission. However, recurrence of the pre-B cell ALL required an unrelated allogeneic cord hematopoietic stem cell transplant (alloHSCT). Post-transplant, the patient developed Graft-Versus-Host Disease (GVHD), which was treated with immunosuppressant therapy for six years until resolution. Fourteen years following the transplant, the patient developed a morbilliform drug eruption secondary to clindamycin. She consequently received prednisone treatment. During the treatment period, the patient developed a new ulcerated and tender nodule on the dorsal aspect of her right hand. Further histopathological biopsy confirmed the diagnosis of C-SCC, which required excision. Ten months following the excision, the patient developed an additional C-SCC nodule on the same right hand, separated by 2.6 cm from the prior C-SCC. She was referred for a ray resection procedure. This case illustrates a patient with multiple risk factors that may have contributed to the continued development of C-SCC. Such risk factors include: a prolonged course of immunosuppressant medications and voriconazole treatment. Additional research is needed to investigate the etiologies and risks of C-SCC development in patients who require a transplant and long-duration immunosuppressive therapy.

## 1. Introduction

Allogenic hematopoietic stem cell transplantation (alloHSCT) increases the risk of developing cutaneous squamous cell carcinomas (C-SCC) [1]. The pathogenesis of C-SCCs in alloHSCT is likely multifactorial and, as in our case, includes compromised immune surveillance, carcinogenic effects of ultraviolet (UV) radiation, and direct pro- and anticarcinogenic effects of drugs [2,3]. Global length of immunosuppression also matters in the progression of C-SCC [4,5]. Age, complexion, and chronic Graft-Versus-Host Disease (GVHD) are additional independent risk factors for C-SCC development in patients after alloHSCT [3,6].

Graft-Versus-Host Disease is a transplantation-associated immunological response mediated by T-lymphocytes found in the graft. These T-lymphocytes recognize the host as foreign and attack multiple tissues, resulting in a systemic inflammatory reaction [7]. GVHD is a multiorgan disease. It notably affects the skin (75% of patients), oral mucosa (51–63% of patients), gastrointestinal tract (22–51% of patients), liver, and eyes [8].

**Figure 1:**
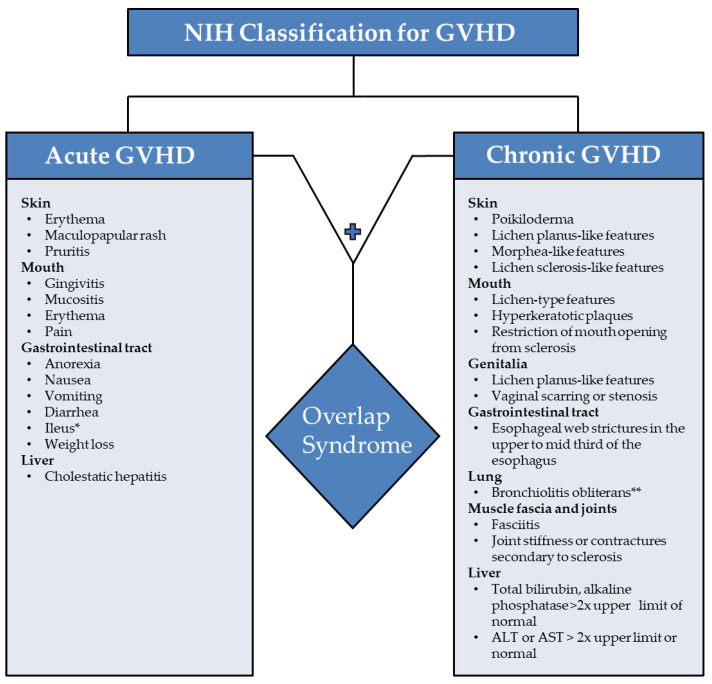
Symptoms of acute and chronic Graft-Versus-Host Disease with overlap syndrome according to NIH classification [9]. Legend: GVHD indicates Graft-Versus-Host Disease; ALT, alanine aminotransferase; AST, aspartate aminotransferase. * Symptoms specific to acute GVHD; ** Requires biopsy to confirm diagnosis.

Acute and chronic GVHD were previously categorized as onset of less than 100 days versus more than 100 days post-transplantation, respectively [9]. However, chronological distinction is no longer utilized. Updated guidelines from the National Institute of Health (NIH) pertaining to clinical and pathological features have been implemented [9]. (Figure 1) The newer classification divides GVHD into the following categories: classic acute GVHD (aGVHD), late aGVHD, and chronic GVHD (cGVHD). Chronic GVHD can arise de novo (also known as classic cGVHD) or as a complication of acute GVHD (also known as overlap syndrome) [10].

Chronic GVHD is diagnosed based on the following NIH consensus criteria [9]:

1. Distinction from acute GVHD;
2. Presence of at least one diagnostic clinical sign of chronic GVHD or presence of at least one distinctive manifestation confirmed by biopsy or other relevant tests (Figure 1);
3. Exclusion of other possible diagnoses.

Skin involvement is the most common finding seen in both chronic and acute GVHD. Many of these skin eruptions are non-malignant [11]. However, patients with GVHD have impaired immunity, which increases the risk for nonmelanoma skin cancer progression. [12] Therefore, suspicious skin lesions seen in patients with GVHD warrant skin biopsy to distinguish between benign cutaneous manifestations and nonmelanoma skin cancers [13,14]. Acute GVHD has not been associated with an elevated risk of basal cell carcinoma (BCC) or squamous cell carcinoma. On the other hand, cGVHD confers a five-fold increase in SCC and a two-fold increase in BCC [15].

Co-infection with human papilloma virus (HPV) increases the risk of developing cutaneous squamous cell carcinoma [16]. This oncogenic process may be mediated by HPV oncogenes E6 and E7, which inhibit the tumor suppressors retinoblastoma (RB) and P53, respectively [17]. Inhibition of RB by E6 upregulates the expression of the tumor suppressor P16 in infected cells [18]. As such, P16 may be used as a surrogate biomarker for HPV-associated squamous cell carcinoma [19]. Despite its aberrant overexpression, the utility of P16 as a surrogate marker for squamous cell carcinoma remains controversial [20]. 

Approximately 5% of all nonmelanoma skin cancers have high-risk clinicopathologic features, including a tumor size ≥ 2 cm, poor tumor differentiation, perineural invasion, depth of invasion, and immunosuppression [21]. We herein report a rare case of a teenage female with two aggressive squamous cell carcinomas on the same hand within a 12-month period treated with Mohs surgery and a fifth ray resection, which can be used when a digital amputation is required [22]. 

## 2. Case Report

A fair-skinned 19-year-old female had pre-B cell acute lymphocytic leukemia at the age of four. Initial remission was achieved with chemotherapy alone, but her leukemia recurred, prompting an unrelated allogeneic cord blood hematopoietic stem cell transplant. Her post-transplant course was complicated by acute followed by chronic sclerotic GVHD. Additionally, she experienced multiple bacterial, viral, and fungal infections. Her chronic GVHD was treated with prednisone (18 months), narrow-band type B ultraviolet (NB-UVB) therapy (18 treatments), type A ultraviolet therapy with psoralen (PUVA) (21 treatments), tacrolimus (26 months), mycophenolate mofetil (8 months), rituximab (3 treatments), intravenous immune globulin (IVIG) (2 treatments), voriconazole (35 days), and photopheresis (1 treatment). Immunosuppressive therapy was continued for a total of six years until her GVHD was quiescent and the therapy was discontinued. 

**Figure 2:**
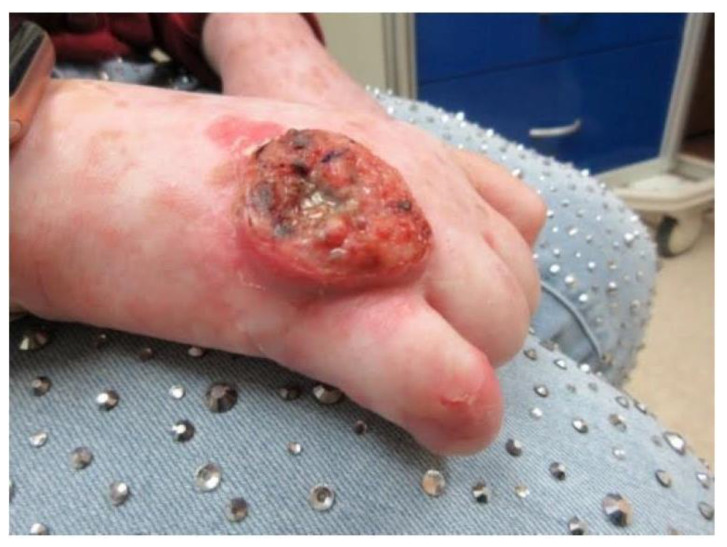
Right dorsal hand with 3.5 cm fungating, ulcerated, and slightly tender plaque with a pushing border.

**Figure 3:**
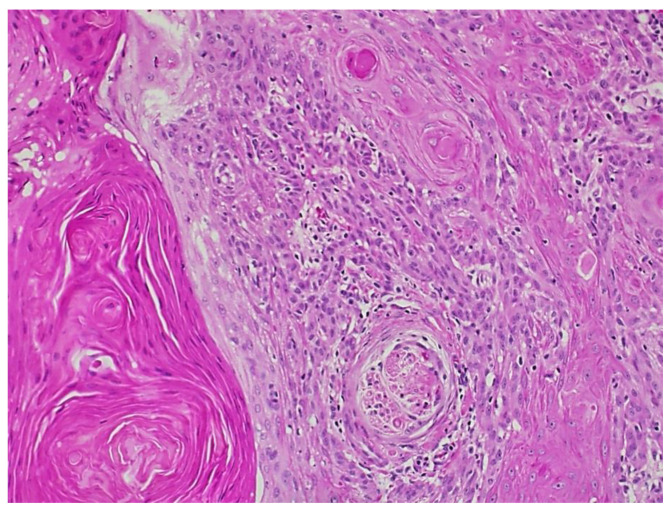
Squamous cell carcinoma with acantholytic features.

Fourteen years after the transplant, the patient was seen in dermatology for a morbilliform drug eruption to clindamycin, which was treated with six weeks of oral prednisone. While being followed for her drug eruption, she developed a rapidly growing 3.5 cm fungating, ulcerated, and slightly tender nodule on her right dorsal hand. (Figure 2) Punch biopsy revealed a cutaneous squamous cell carcinoma with acantholytic features. (Figure 3) Immunohistochemical staining for P16 was negative. This test was performed to rule out co-infection with HPV.

The patient underwent Mohs surgery. The tumor was cleared after two stages of Mohs surgery. The tumor superficially invaded the tendon sheath of the right fourth extensor digitorum communis. Intraoperatively, orthopedic hand surgery was consulted and assisted with the final stage of severing the fourth extensor tendon. After six months of secondary intention healing and placement of partial split thickness skin grafts, the wound had completely re-epithelialized, though with substantial scar contraction.

**Figure 4:**
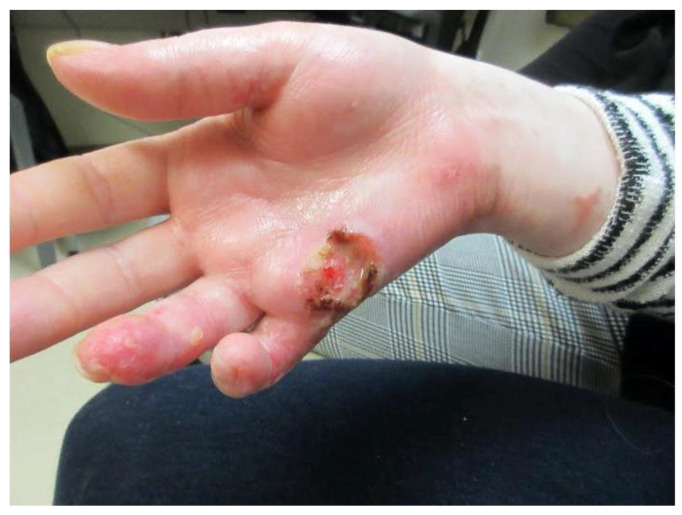
Right ventral hand with 2.4 cm, fissured, indurated, erythematous, ulcerated, and depressed plaque.

**Figure 5:**
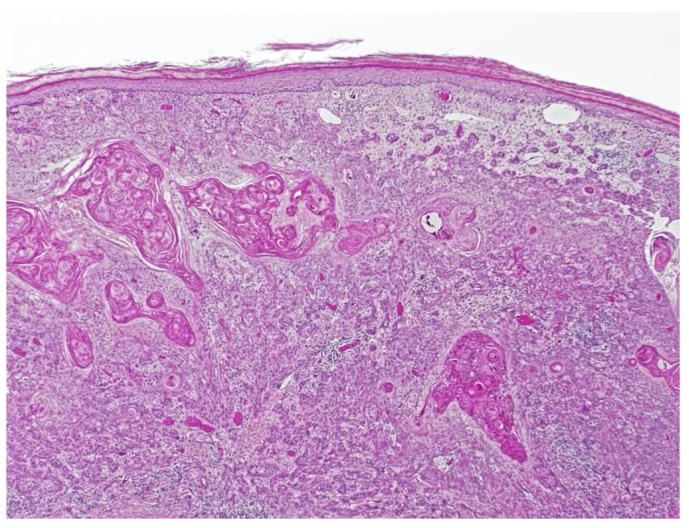
Squamous cell carcinoma with perineural invasion of greater than 0.1 mm.

Ten months after Mohs surgery, the patient presented with a 2.4 cm fissured, indurated, erythematous, ulcerated plaque on the right ventral ulnar palm. (Figure 4) This was separated by 2.6 cm of normal skin from the scar from the prior C-SCC on the right hand. Punch biopsy revealed squamous cell carcinoma with perineural invasion involving a 0.1 mm cutaneous nerve. (Figure 5) With her scar contracture and functional impairment in that hand, she was referred to orthopedic oncology. After discussion of risks and benefits for excision with secondary intent healing versus a ray resection, the latter was agreed upon. 

A lymph node exam was challenging in this patient with her sclerotic Graft-Versus-Host Disease. Therefore, a positron emission tomography–computed tomography was obtained, which indicated no local or distant hypermetabolic foci concerning for metastatic disease.

## 3. Discussion

The literature on cutaneous squamous cell carcinoma in pediatric patients following allogeneic hematopoietic stem cell transplant (alloHSCT) remains limited. Although most studies have been conducted on adult patients (18 years and older), investigational conclusions may tentatively apply to the pediatric population. Table 1 lists the various host-associated and transplant-associated risk factors which correlate with SCC.

After the allogeneic hematopoietic stem cell transplantation, the patient developed acute followed by chronic GVHD. Her treatment consisted of tacrolimus, mycophenolate mofetil, prednisone, voriconazole, IVIG, PUVA, NB-UVB, IVIG, and photoperesis for a total of six years. Following the completion of the regimen, she acquired two consecutive cutaneous squamous cell carcinomas on her right hand. The patient had multiple predisposing factors which put her at risk for developing C-SCC. Such factors include fair complexion, history of cGVHD, voriconazole use, and prolonged immunosuppressive therapy (Table 1).

Chronic GVHD is a major complication of bone marrow transplants seen in more than 50% of alloHSCT cases [30,31]. Multiple reports have identified cGVHD as an independent risk factor for oral and skin squamous cell cancers in alloHSCT recipients [1,3,29,32]. Curtis et al. revealed that cGVHD in alloHSCT patients conferred a three-fold increase in the risk of acquiring SCC compared to those with no history of cGVHD [1]. The majority of cGVHD cases arise as a complication of acute GVHD (a condition of inflammation and immunodeficiency). Immunodeficiency and inflammation invoke mutations that halt DNA repair processes, which may contribute to squamous cell carcinogenesis [1,33,34,35,36]. Therefore, the patient’s history of acute followed by chronic GVHD inceased her risk of developing SCC [1]. 

Furthermore, the six-year history of immunosuppressive treatment is an additional risk factor for C-SCC in this patient. Immunosuppressive medications may be considered as a potential risk factor in the progression of C-SCC. The duration of immunosuppression directly correlates with an elevated risk of C-SCC in patients with GVHD [1]. Curtis et al. revealed 24 months or more of immunosuppressive therapy, particularly with azathioprine (*p* < 0.001), demonstrated an eight-fold risk of SCC compared to patients who had not undergone chronic GVHD therapy [1]. However, mycophenolate mofetil and tacrolimus have a significantly lower risk of SCC in comparison to azathioprine [37]. 

Other components of the patient’s treatment include: short-term systemic corticosteroids and phototherapy. The patient received prednisone (a synthetic glucocorticoid) for 18 months [38]. Despite its immunosuppressive properties, long-term systemic corticosteroids use does not increase the risk of SCC [39].

Additionally, the patient was exposed to NB-UVB and PUVA treatments. NB-UVB phototherapy is a widely used second-line treatment for GVHD in pediatric patients. [40,41] Multiple studies revealed no increased risk of skin cancer among patients who received NB-UVB [41,42,43]. In contrast, 350 treatments of PUVA but not fewer than 150 treatments increase the risk of SCC [44]. Therefore, with only 21 PUVA treatments, our patient maintained a low risk of SCC development.

The prolonged immunosuppressed state predisposed the patient to multiple fungal and bacterial infections. Consequently, she required a 35-day course of voriconazole. Voriconazole is an effective and widely used antifungal medication in transplant and immunodeficient patients [45,46]. However, long-term use of this medication increases the risk of photosensitivity and nonmelanoma skin cancer [26,47,48]. Voriconazole undergoes hepatic metabolism to voriconazole-N-oxide (VNO). VNO sensitizes keratinocytes to UVA and generates reactive oxygen species (ROS), which causes oxidative DNA damage [49]. Continuous accumulation of damaged DNA in cutaneous tissues increases the risk of skin cancer [49,50]. Kuklinski et al. reported that voriconazole use (treatment interval not specified) is strongly associated with SCC in alloHSCT patients [26]. Other studies demonstrated a dose-dependent increase in SCC in voriconazole-exposed alloHSCT recipients. Prior research found a 3.0% increased risk per 30-day exposure and 5.6% increased risk per 60-day exposure at a standard dose of 200 mg twice daily [47,48]. Therefore, the 35-day course of voriconazole treatment puts the patient at moderate risk for developing C-SCC. 

Additionally, HPV co-infection was considered as a risk factor for the development of squamous cell carcinoma in this patient. As such, immunohistochemical staining for p16 was ordered, which yeilded negative results. As noted, NB-UVB, PUVA, mycophenolate mofetil, and prednisone have a negligible risk for SCC. Long-term systemic use of tacrolimus has been shown to increase the risk of SCC in organ transplant patients [51], but given the brief duration of tacrolimus treatment in this patient, it is less likely a contributing factor for her development of SCC. Based on the evidence in the current literature, the two plausible risk factors applicable to our patient include her treatment history of cGVHD and voriconazole use. While the risks with voriconazole have been relatively characterized, further research is recommended to understand the relationship between GVHD therapy and the progression of SCC. 

## 4. Conclusions

We herein report a unique case of a patient who developed C-SCC following long-term immunosuppressive therapy for the treatment of GVHD. While the exact mechanism of C-SCC progression in this patient remains unknown, she had multiple predisposing risk factors. Her C-SCC development was likely triggered by post-transplant factors, most notably all listed GVHD risk factors and voriconazole usage. 

Immunosuppressed patients should receive a full-body dermatological exam every six to twelve months, if possible, in a high-risk cutaneous oncology clinic. These screening exams are crucial to prevent and detect dermatological neoplasms at early stages. 

Multidisciplinary discussions are crucial as tumors may present with aggressive features and standard closures may be challenging in patients with widespread sclerosis. Additional investigations are essential to understand the pathomechanisms and risk factors related to SCC development among transplanted patients who require long-duration immunosuppressive therapy. Such research may improve the standard of care and mitigate risks for transplanted patients who undergo long-term immunosuppressive therapy.

## Figures and Tables

**Table 1:** List of transplant-associated and host-associated risk factors which correlate with increased risk of squamous cell carcinoma *.

**Transplant-associated risk factors include:**
Chronic lymphocytic leukemia [23,24]; Donor source [1,5,25]; Conditioning regimen [3,5,25]; Voriconazole exposure [26,27]; Immunosuppression [1]; Cumulative days of immunosuppression [1]; Acute GVHD [3,6,28]; Chronic GVHD [1,3,5,6,15,28,29].
**Host-associated risk factors include:**
Age [3,5,6,26,28]; Sex [5,26,28,29]; Pigmentary phenotype [26]; Radiation therapy (including UVA-based phototherapy) [23,24]; UVR exposure (outdoor occupation and photodamaged skin) [23,24]; Prior skin cancer [26].

* List is not exhaustive.

## Data Availability

Not applicable.
